# Disruption of *Smad4* Expression in T Cells Leads to IgA Nephropathy-Like Manifestations

**DOI:** 10.1371/journal.pone.0078736

**Published:** 2013-11-04

**Authors:** Hiroyuki Inoshita, Byung-Gyu Kim, Michifumi Yamashita, Sung Hee Choi, Yasuhiko Tomino, John J. Letterio, Steven N. Emancipator

**Affiliations:** 1 Department of Pathology, Case Western Reserve University, Cleveland, Ohio, United States of America; 2 Department of Pediatrics, Case Western Reserve University, Cleveland, Ohio, United States of America; 3 Division of Nephrology, Department of Internal Medicine, Juntendo University Faculty of Medicine, Bunkyo-ku, Tokyo, Japan; 4 Pathology and Laboratory Medicine Service, Louis Stokes Department of Veterans Affairs Medical Center, Cleveland, Ohio, United States of America; 5 Department of Pathology, University Hospitals Case Medical Center, Cleveland, Ohio, United States of America; Institut national de la santé et de la recherche médicale (INSERM), France

## Abstract

The link between glomerular IgA nephropathy (IgAN) and T helper 2 (Th2) response has been implicated, however, the mechanisms are poorly defined because of the lack of an appropriate model. Here we report a novel murine model characterized by lineage-restricted deletion of the gene encoding MAD homologue 4 (Smad4) in T cells (*Smad4^co/co;Lck-cre^*). Loss of *Smad4* expression in T cells results in overproduction of Th2 cytokines and high serum IgA levels. We found that *Smad4^co/co;Lck-cre^* mice exhibited massive glomerular IgA deposition, increased albumin creatinine ratio, aberrant glycosylated IgA, IgA complexed with IgG1 and IgG2a, and polymeric IgA, all known features of IgAN in humans. Furthermore, we examined the *β*1, 4-galactosyltransferases (*β*4GalT) enzyme which is involved in the synthesis of glycosylated murine IgA, and we found reduced *β*4GalT2 and *β*4GalT4 mRNA levels in B cells. These findings indicate that *Smad4^co/co;Lck-cre^* mice could be a useful model for studying the mechanisms between IgAN and Th2 response, and further, disruption of Smad4-dependent signaling in T cells may play an important role in the pathogenesis of human IgAN and contributing to a Th2 T cell phenotype.

## Introduction

IgA nephropathy (IgAN) is the most common form of glomerulonephritis in the world. This disease leads to progressive renal failure in a substantial proportion of patients. Despite an expanding international research effort over 40 years, the mechanisms of pathogenesis are still obscure. The hallmarks of this disease are hematuria, variable degrees of proteinuria, low grade proliferative glomerulonephritis, and immune deposition of predominantly IgA in the renal mesangium. Dysregulated circulating IgA is often found in patients with IgAN; notably, the elevated levels of IgA in serum are aberrantly glycosylated and biased towards higher molecular weight isoforms, and bound in complexes with IgG that are specific for truncated glycans [1]–[4].

Aberrant T helper 2 (Th2) cytokine production has been implicated in the pathogenesis of IgAN. Compared to other forms of glomerulonephritis, IgAN is more common in industrialized nations; this suggests that less exposure to microorganisms results in decreased T helper 1 (Th1) response and increased Th2 response [5], [6]. Moreover, patients with IgAN exhibit bias toward Th2 cytokine production during disease exacerbation, but heightened secretion of Th1 cytokines during remission [7], [8]. Both human and murine B cells *in vitro* produce higher aberrant glycosylated IgA levels in response to a mixture of recombinant interleukin (IL)-4 and IL-5 (Th2 cytokine) compared to control cultures [9], [10]. mRNA levels of both core 1 *β*1,3-galactosyltransferase (*β* 3GalT) and its molecular chaperone Cosmc, as well as *β*3GalT enzymatic activity are down-regulated by recombinant IL-4 in human B cells [11]. Thus, this suggests that skewed Th2 cytokine production leads to elevated levels of abnormally glycosylated IgA in the serum of patients with IgAN.

Among other cytokines, transforming growth factor-β (TGF-β) powerfully regulates the activation, differentiation, and function of T cells [12], [13]. Upon binding to its receptor, TGF-β activates the transcription factors Smad2 and Smad3; activated Smad2 and Smad3 complex with Smad4, and subsequently translocates into the nucleus [14]. Recently, we generated conditional knockout mice in which the gene encoding *Smad4* is deleted selectively in T cells (*Smad4^co/co;Lck-cre^*) by crossing mice homozygous for a *Smad4* allele flanked with the *lox* sequence with mice expressing a transgene encoding a *Cre* recombinase driven by the *lck* promoter [15]. Interestingly, these *Smad4^co/co;Lck-cre^* mice show higher serum IgA levels than WT controls, and their T cells produce abundant Th2 cytokines (e. g. IL-4, IL-5, IL-6 and IL-13) when stimulated *in vitro*. In this study, we investigated characteristics of *Smad4^co/co;Lck-cre^* mice in terms of IgAN to elucidate whether the proclivity to produce Th2 cytokines due to the selective loss of Smad4 signaling in T cells could lead to IgAN-like phenotype.

## Materials and Methods

### Ethics Statement

All animal studies have been approved by Case Western Reserve University (Cleveland, OH) Institutional Animal Care and Use Committee (Protocol Number: 2007-0043) and maintained in the Animal Resource Center at Case Western Reserve University. Animal procedures were conducted in compliance with National Institutes of Health Guidelines.

### Animals

The generation of the *Smad4^co/co;Lck-cre^* female mice on a C57BL/6×SvEv129×FVB background was described previously (15). In brief, conditional *Smad4* allele (*Smad4^co/co^*) mice [16] were bred with Lck-Cre (Taconic, Petersburgh, NY) mice. F1 offspring heterozygous for the conditional allele (*Smad4^+/co^*) were inbred to generate homozygous *Smad4* conditional mice (*Smad4^co/co^*) with Cre recombinase transgenes. Age-matched C57BL/6×SvEv129×FVB female mice were used as controls. After mice were sacrificed, blood samples were obtained by cardiac puncture and the kidneys harvested and decapsulated. The left kidney was cut perpendicular to the long-axis and one half of the kidney was embedded in optimal cutting temperature (OCT) compound (Tissue-Tek; Sakura Finetek, Torrance, CA) followed by freeze on dry ice, while the second half was fixed in 10% neutral-buffered formalin followed by paraffin embedding.

### Creatinine measurement

For determination of urinary creatinine, alkaline picrate method was used. Standards (Sigma-Aldrich, Saint Louis, MO) and 20 µl of diluted urines (1∶10) were added into a 96-well microtiter plate. Alkaline picrate solution (10.8 mM picric acid, 29 mM sodium borate, 167 mM NaOH, 1.67% SDS (w/v)) was incubated for 10 min at RT, and absorbance was read at 490 nm. After the measuring, 60% acetic acid was added into all wells and left for 8 min at RT. The absorbance was read at 490 nm again, and then subtracted from the first absorbance.

### ELISA

IL-4, IL-5, IL-13, albumin, IgA, IgM, IgG1, IgG2a, IgG1-IgA complex, IgG2a-IgA complex, and hemoglobin were measured by performing sandwich ELISA assays. Quantitative ELISA kits were purchased from Bethyl (Montgomery, TX) for albumin, IgA, IgM, IgG1, and IgG2a measurement and from R&D Systems (Minneapolis, MN) for IL-4, IL-5, and IL-13 measurement, respectively. The assays were developed according to the recommendation of the manufacturers. To measure IgG1-IgA complex and IgG2a-IgA complex, goat anti mouse IgG1antibody and IgG2a antibody (Bethyl) were used as coating antibody, respectively [17]. Goat anti-mouse IgA antibody was used as detection antibody. Hemoglobin ELISA was carried out as described before [18]. A common protocol of these sandwich ELISAs is as follows; Wells of a 96-well microtiter plate was coated with coating antibody in 100 µl of coating buffer (100 mM NaHCO_3_, 100 mM Na_2_CO_3_, pH9.2) for 1h at room temperature (RT). After blocking with blocking buffer (50 mM Tris-HCl, 140 mM NaCl, 1% (w/v) BSA, pH 8.0) for 1h at RT and washing with washing buffer (50 mM Tris-HCl, 140 mM NaCl, pH 8.0), 100 µl of diluted samples and standards in sample buffer (50 mM Tris-HCl, 140 mM NaCl, 1% BSA (w/v), 0.05% (v/v) Tween 20, pH 8.0) were transferred to assigned wells, and incubated for 1 h. The wells were washed, and then HRP-conjugated detecting antibody was added to each well for 1 h at RT. Color development was performed by utilizing TMB substrate (R&D system). To stop the reaction, 100 µl of H_2_SO_4_ (Sigma-Aldrich) was applied to each well. The absorbance at 450 nm was determined using a microplate reader.

### Lectin ELISA

Glycosylation of IgA was assessed by lectin ELISA, as described previously, with minor modifications [19]. In brief, wells of a 96-well microtiter plate were coated with 1 µg of goat anti-mouse IgA (Bethyl) in 100 µl of coating buffer. Uncoated binding sites were blocked with 250 µl of blocking buffer at RT for 1 h. After washing with wash buffer, sera diluted with sample buffer were incubated in the wells for 1 h at RT in duplicate. Following incubation and washing, 0.8 µg of biotinylated *Sambucus nigra* (SNA; Vector, Burlingame, CA) or 2 µg of biotinylated *Risinus communis* agglutinin-I (RCA; Vector) in 100 µl of sample buffer was incubated in the wells for 1 h at RT. After additional washing, 1∶ 1000 and 1∶5000 dilution of avidin biotin complex (Vector) were added to the wells for SNA and RCA ELISA, respectively. The incubation was performed for 1 h at RT. After each step, development was performed utilizing 100 µl/well of TMB substrate and stopped by addition of H_2_SO_4_.

### IgA Western blot analysis

Serum (0.25 µl) was loaded with SDS loading buffer and resolved by 8% SDS-polyacrylamide gel electrophoresis (PAGE), transferred onto a PVDF membrane (Millipore, Billerica, MA). The membrane was blocked with 5% BSA solution for 1 h at RT, then immunoblotted with HRP-conjugated goat anti-mouse IgA for 1 h at RT. Detection by enzyme-linked chemiluminescence was performed according to the manufacturer's protocol (ECL; GE Healthcare, Pittsburgh, PA).

### Immunofluorescence

Murine kidneys embedded in OCT were cut into 3 to 4 µm sections, and collected on clean glass slides. The sections were fixed in acetone at 4°C and rinsed in phosphate buffered isotonic saline (PBS, pH7.4) 3 times for 5 min. Direct IF was performed using FITC-conjugated goat anti-mouse IgG, IgA, and IgM (Southern Biotech, Birmingham, AL). Indirect IF was performed using rat monoclonal antibody for mouse C3 (Abcam), and then FITC-conjugated polyclonal goat anti-rat IgG (Abcam) was used as the secondary antibody. All sections were incubated with these antibodies for 1 h at RT. Observations were recorded with a Leica DMLB microscope for IF documentation.

### Electron microscopy

After excision of the kidney, the capsule was removed and the kidney was sliced into small tissue samples (approximately 2 mm^3^). These samples were further treated with 2% glutaraldehyde and 1% osmium tetroxide, dehydrated in graded ethanol, and embedded in epoxy resin. Ultrathin sections were examined using a transmission electron microscope (HT7700 120 kV; Hitachi, Tokyo, Japan).

### Cell isolation and culture

Single cell suspensions were prepared from spleens of naïve WT and the *Smad4^co/co;Lck-cre^* female mice by macerating organs and filtering through nylon mesh (40 µm diameter). Erythrocytes were lysed using ACK lysis buffer (BioWhittaker, Walkersville, MD) and cells were washed twice in RPMI 1640 complete medium (RPMI 1640 supplemented with 10% heat-inactivated FBS, 50 µM 2-ME, penicillin, and streptomycin). Viable cells were counted using trypan blue exclusion and a hemacytometer. For cytokine production measurements, Pan T cells were isolated from spleen and lymph node using a Pan T Cell Isolation Kit (Miltenyi Biotec, Auburn, CA) according to the manufacturer's instruction (purity greater than 95%). 5×10^4^ spleen T cells or lymph nodes T cells were stimulated in 0.2 ml complete media per well in 96-well plates coated with 2 µg/ml anti-CD3 mAb and 1 µg/ml anti-CD28 mAb (BD Biosciences, San Jose, CA). After 2 days, T cells supernatants were collected and used for ELISA assay. For mRNA levels of β4GalT1 to 7, splenic B cells were isolated using B Cell Isolation Kit, CD19 mAb-coated microbeads (Miltenyi Biotec), according to the manufacturer's instructions. The purity of cells was usually greater than 95%.

### Isolation of RNA and real-time reverse transcription polymerase chain reaction (RT-PCR)

Trizol reagent (Invitrogen, Grand Island, NY) was used for the isolation of total RNA from the spleen B-cells. First-strand cDNA was synthesized using the High-Capacity cDNA Reverse Transcription kit (Applied Biosystems, Grand Island, NY). The reaction was carried out at 25°C for 10 min, 37°C for 120 min and 85°C for 5 min on Px2 Thermal Cycler (Thermo electron corporation, Waltham, MA). Real-time reverse transcription polymerase chain reaction (RT-PCR) was carried out on a IQ SYBR Green (Bio-Rad Laboratories, Hercules, CA) using C1000 Thermo cycler (Bio-Rad Laboratories). Glyceraldehyde 3-phosphate dehydrogenase (GAPDH) was used as the control gene for normalization of mRNA expression. The sequences of the primers used in this study are provided in Table 1.

**Table 1:** PCR Primers.

	Forward primer	Reverse primer	product size (bp)
βGalT1	AATGATCCGGCATTCAAGAG	CGATGTCCACTGTGATTTGG	167
βGalT2	AGCCAGCAGCAGTACCAACT	TGAGGTGAATTCGATGACCA	163
βGalT3	GGAACGTTTAACAGGGCAAA	GTACGGGAGGCTGTATCCAA	192
βGalT4	ACCTGGTGCCTGAGAATGAC	GGAGCTCAACCCTGAGTCTG	222
βGalT5	GAGCGGCCTGACTGTAGAAC	AGAGCGTACCTGCCAAGAAA	204
βGalT6	TTTTTCCTGCACCTGATTCC	CCGATCATTTTCAGGCAGAT	192
βGalT7	AGAGGATCCAGCACCACATC	AGCCATAGTCCAGCTCCTCA	165
GAPDH	ACCACAGTCCATGCCATCAC	CACCACCCTGTTGCTGTAGCC	450

βGalT, β1,4 galactosyltransferase; GAPDH, Glyceraldehyde 3-phosphate dehydrogenase.

## Results

### Th2 cytokine production by T cells from *Smad4^co/co;Lck-cre^* mice

**Figure 1:**
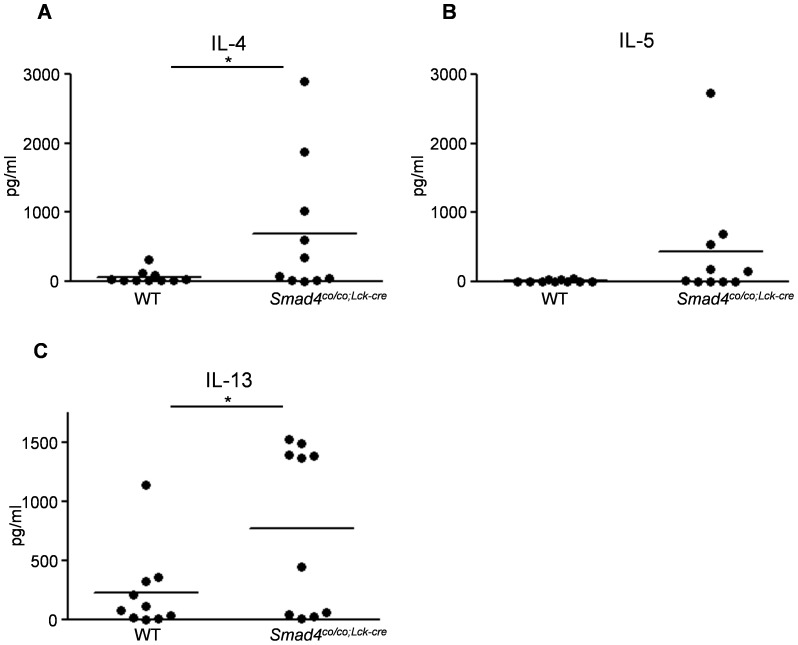
Loss of Smad4 signaling in T cells induces increased Th2 cytokine production by T cells. The levels of (A) IL-4 and (C) IL-13 in T cell supernatant were significantly increased in *Smad4^co/co;Lck-cre^* mice (n = 10) compared with WT mice (n = 10). *Smad4^co/co;Lck-cre^* mice tended to have higher (B) IL-5 levels than WT mice (P = 0.066). Data are expressed as the mean±SEM; P values were calculated with Student's *t* test.*P<0.05

It has been suggested that T cells lacking Smad4 are implicated in the secretion of copious amounts of IL-4, IL-5, and IL-13, however, the levels of these cytokines in T cell-specific Smad4 deficient (*Smad4^co/co;Lck-cre^*) mice have not been determined. We measured the levels of IL-4, IL-5, and IL-13 in supernatants of T cells from *Smad4^co/co;Lck-cre^* and aged-matched wild type (*Smad4^+/+;Lck-cre^* ;designated WT) mice. The levels of Th2 cytokines, especially IL-4 and 13, secreted by T cells from *Smad4^co/co;Lck-cre^* mice were significantly increased as compared to aged-matched WT mice (Figure 1).

### Levels of Th2 cytokines in serum of *Smad4^co/co;Lck-cre^* mice

**Figure 2:**
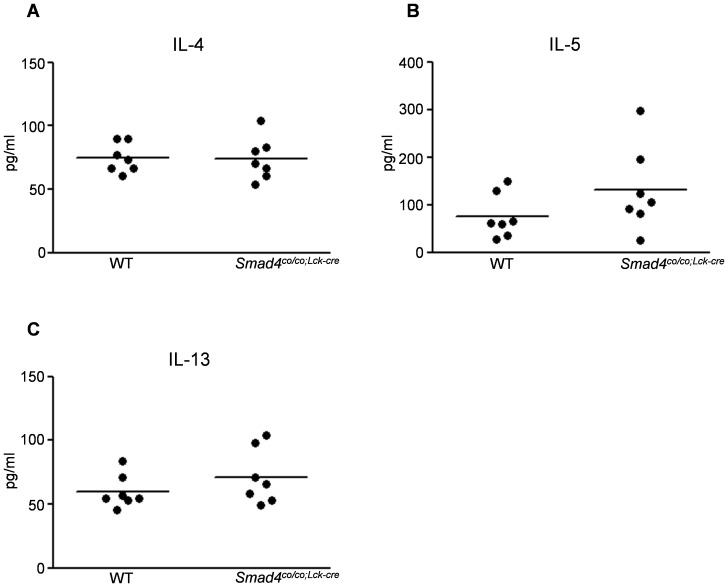
Levels of Th2 cytokines in serum of *Smad4^co/co;Lck-cre^* mice. The levels of IL-4, IL-5, and IL-13 in the serum from *Smad4^co/co;Lck-cre^* (n = 7) and WT (n = 7) mice are were determined by ELISA. No significant differences were observed between the two groups. Data are expressed as the mean±SEM; P values were calculated with Student's *t* test.

To investigate whether the lacking Smad4 in T cells affects serum levels of Th2 cytokines, IL-4, IL-5, and IL-13 in the serum from *Smad4^co/co;Lck-cre^* were measured using ELISA. There were no significant differences in the serum levels of the all Th2 cytokines between *Smad4^co/co;Lck-cre^* and controls (Figure 2).

### Levels of immunoglobulins in serum of *Smad4^co/co;Lck-cre^* mice

**Figure 3:**
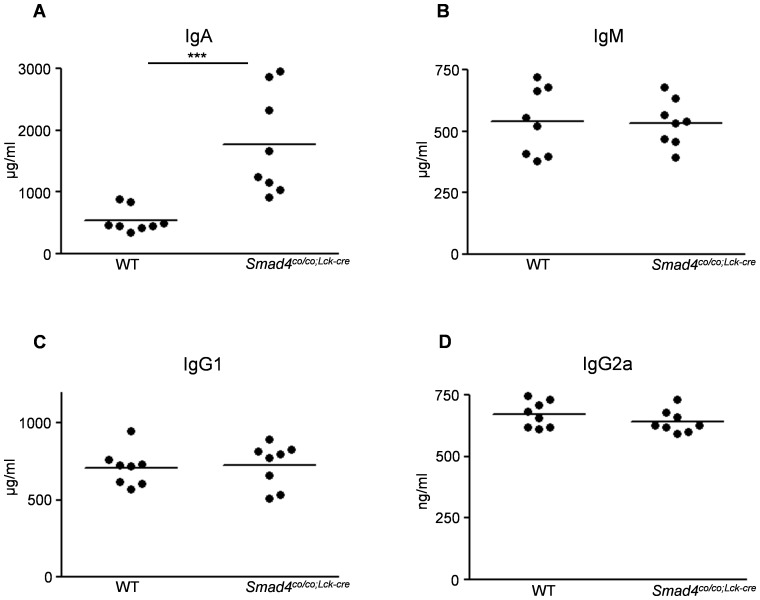
Loss of Smad4 signaling in T cells induces high serum levels of IgA. The serum levels of IgA, IgM, IgG1, and IgG2a were determined using ELISA. (A) Markedly increased serum levels of IgA in *Smad4^co/co;Lck-cre^* mice (n = 8) compared with WT mice (n = 8) (1773±294.3 vs 545.2±71.39). The results from serum levels of (B) IgM, (C) IgG1, and (D) IgG2a showed no significant differences between the two groups. Data are expressed as the mean±SEM; P values were calculated with Student's *t* test. ***P< 0.001

As reported previously (15), the levels of IgA in the serum from *Smad4^co/co;Lck-cre^* at 3 months of age were dramatically increased compared with WT mice (Figure 3A). No significant differences in circulating IgM, IgG1, and IgG2a levels were observed between *Smad4^co/co;Lck-cre^* mice and WT mice at 3 months of age (Figure 3, B-D).

### Predominant IgA deposition in glomeruli and glomerular function of *Smad4^co/co;Lck-cre^* mice

**Figure 4:**
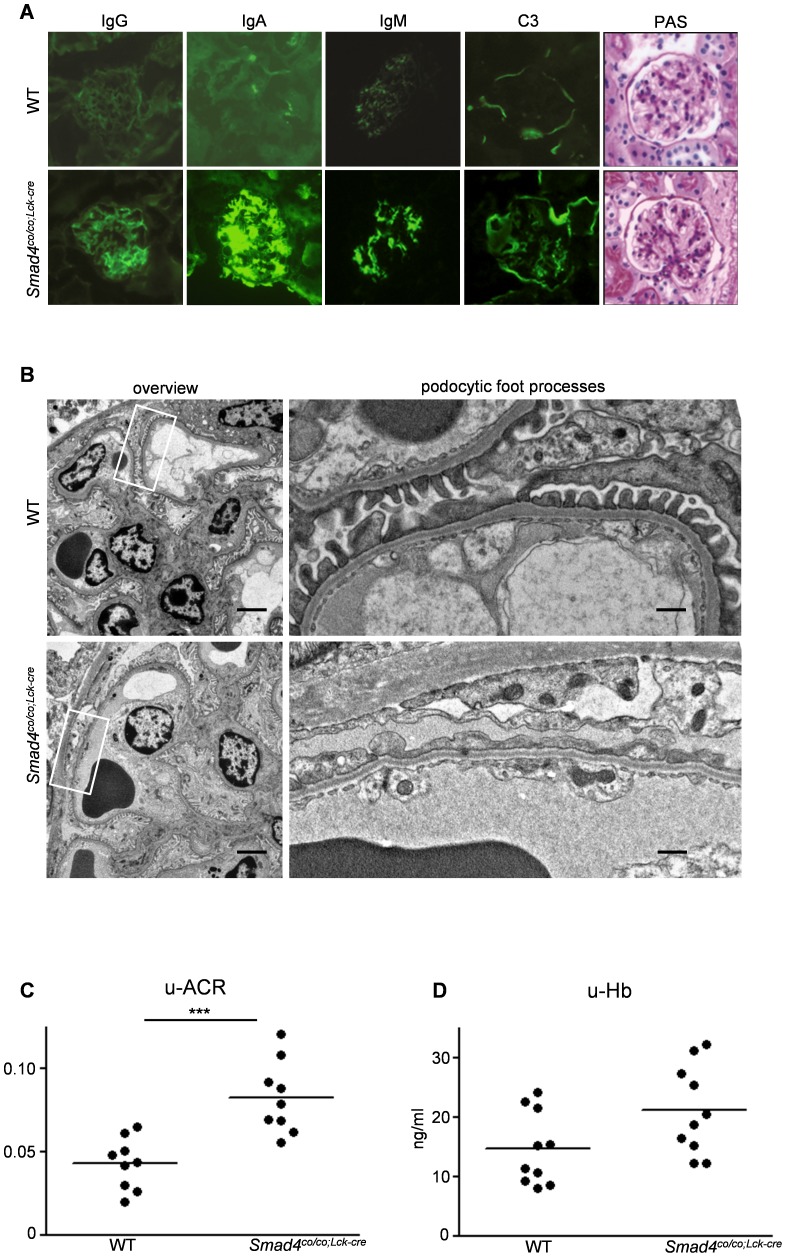
Deletion of *Smad4* in T cell induced predominant IgA deposition in glomeruli and proteinuria. (A) Immunofluorecence studies showed glomerular deposition of predominantly IgA, with lesser amounts of IgG and IgM deposition in *Smad4^co/co;Lck-cre^* mice compared with WT mice at 3 months of age. C3 deposition was occasionally observed in both mesangial areas and Bowman's capsule in *Smad4^co/co;Lck-cre^* mice, but was restricted to Bowman's capsule in WT mice. PAS staining showed no significant difference between WT and *Smad4^co/co;Lck-cre^* mice. Original magnification; ×400 (B) Electron microscopic overview (left panels) and detail of podocytic foot processes (right panels) for *Smad4^co/co;Lck-cre^* (bottom panels) and WT (top panels) mice. Podocyte foot process effacement was partially observed in glomeruli from *Smad4^co/co;Lck-cre^* mice compared to WT mice. Scale bars: 5 µm (left panels); 0.5 µm (right panels). (C) Increased albumin creatinine ratio (ACR) was observed in urines of *Smad4^co/co;Lck-cre^* mice (n = 9) compared with WT mice (n = 9) (0.043±0.005 vs 0.083±0.007). (D) There is no significant difference in the urinary hemoglobin between WT (n = 10) and *Smad4^co/co;Lck-cre^* mice (n = 10) (14.7± 1.94 vs 21.2±2.36). Data are expressed as the mean±SEM; P values were calculated with Student's *t* test. ***P< 0.001

By immunofluorescence, intense IgA deposits were detected in the glomerular mesangium in most *Smad4^co/co;Lck-cre^* mice, whereas trivial IgA deposits were found in WT mice (Figure 4A). Mesangial deposits of IgG and IgM were also found in some *Smad4^co/co;Lck-cre^* mice, but only a few controls had meaningful deposits. Weak C3 deposits in mesangial areas and Bowman's capsule were detected in both *Smad4^co/co;Lck-cre^* and WT mice. The distribution of glomerular IgG, IgA, IgM, and C3 deposits is shown in Table 2. Moderate (++) or intense (+++) IgA was detected in approximately 89% of glomeruli in Smad4*^co/co;Lck-cre^* mice, whereas no (–) or weak (+) signals were detected in 94% of WT mice. Significant (> +) IgM was present in 61% of *Smad4^co/co;Lck-cre^* mice, but in only 13% of WT mice; IgG and C3 deposition was less pronounced. By light microscopic examination of periodic acid-Schiff (PAS) stained sections, the kidneys of *Smad4^co/co;Lck-cre^* mice exhibited no marked abnormalities (Figure 4A). Specifically, mesangial proliferation, expansion of mesangial matrix, and other glomerular (or extraglomerular) changes were not detected in *Smad4^co/co;Lck-cre^* mice relative to WT mice, at 3 or 8 months of age. Hematoxylin and eosin (HE)-stained sections of small intestine and colon from both *Smad4^co/co;Lck-cre^* and WT mice at 3 months of age showed no obvious intestinal inflammation (Figure S1). To identify electron-dense deposit (EDD) and podocyte damage in glomeruli at the ultrastructure level, transmission electron microscopic analysis was performed. A typical EDD, which has uniform electron density without specific structure, could not be found in glomeruli from both groups (Figure 4B). In *Smad4^co/co;Lck-cre^* mice, glomerular basement membrane was slightly thinned, and foot processes were effaced in a part of capillarys (Figure 4B). Excretion of albumin in the urine, especially when expressed as an albumin to creatinine ratio (ACR), was significantly higher in *Smad4^co/co;Lck-cre^* mice than in controls (Figure 4C). Although there is no statistical difference between *Smad4^co/co;Lck-cre^* mice and controls, there was a trend toward an increase in urinary hemoglobin (as a measure of hematuria) in *Smad4^co/co;Lck-cre^* mice when compared to controls (Figure 4D).

**Table 2:** Degree of glomerular deposition in kidneys.

	WT				*Smad4^co/co;Lck-cre^*			
	IgG	IgA	IgM	C3	IgG	IgA	IgM	C3
+++	0	0	0	0	0	21	5	2
++	4	3	7	1	17	27	28	9
+	13	27	22	41	24	5	10	36
−	37	24	25	12	13	1	11	7

### Characteristics of circulating IgA in serum of *Smad4^co/co;Lck-cre^* mice

**Figure 5:**
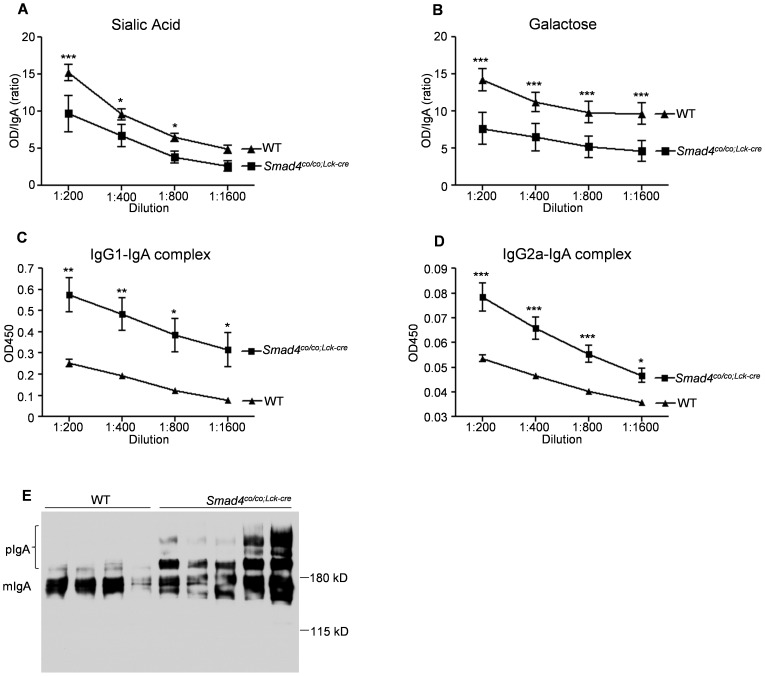
Characteristics of circulating IgA in mice lacking Smad4 in T-cells are similar to those in patients with IgA nephropathy. (A and B) Aberrant glycosylation of circulating IgA from *Smad4^co/co;Lck-cre^* mice (n = 6, black square) and WT mice (n = 6, black triangle) was examined by lectin ELISA. Both (A) sialic acid and (B) galactose on circulating IgA from the mutants were lower than those from controls. Data are expressed as the ratios of specific lectin-binding IgA to total IgA. (C and D) *Smad4^co/co;Lck-cre^* mice (n = 6, black square) exhibit an increased in both (C) IgG1-IgA complexes and (D) IgG2a-IgA complexes in the circulation, compared with WT mice (n = 6, black triangle). Data are expressed as mean±SEM; P values were calculated with *t* test or Bonferroni post test. ***P<0.001, **P<0.01, and *P<0.05 (E) The predominance of polymeric IgA was observed in serum from *Smad4^co/co;Lck-cre^* mice, but not in WT mice, as determined by Western blot.

We analyzed the pattern of glycosylation of IgA; the relative levels of terminal sialic acid (Figure 5A) and terminal or penultimate galactose (Figure 5B) on serum IgA from *Smad4^co/co;Lck-cre^* mice significantly decreased compared with those on the IgA from WT mice. By sandwich ELISA, the levels of complexes of IgG1-IgA and IgG2-IgA in serum from *Smad4^co/co;Lck-cre^* mice were found to be significantly higher than those in WT mice (Figure 5, C and D). Finally, Western blotting revealed that the circulating IgA in *Smad4^co/co;Lck-cre^* mice is predominantly polymeric, whereas most of IgA from WT mice is monomeric (Figure 5E).

### mRNA levels of β4GalTs in B-cells from *Smad4^co/co;Lck-cre^* mice

**Figure 6:**
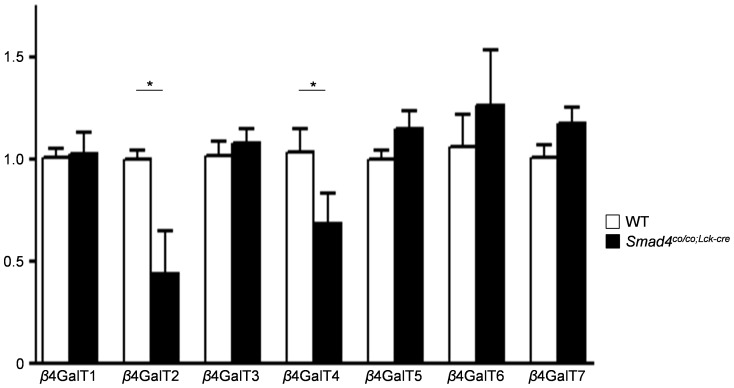
RT-PCR analysis of *β*4GalT mRNAs in *Smad4^co/co;Lck-cre^* mice. mRNA was isolated from purified B-cells (>95% CD19+), and RT-PCR was performed using primers specific for *β*4GalT1 to 7, and GAPDH. *β*4GalT 2 and 4 mRNA expression in *Smad4^co/co;Lck-cre^* mice (n = 4, black column) were significantly reduced relative to controls (n = 4, white column). All samples were normalized to GAPDH levels. Data are expressed as mean±SEM; P values were calculated with *t* test. *P<0.05

In mice, *β*1, 4-galactosyltransferases (*β*4GalT) are involved in the synthesis of IgA glycosylation [20], [21], and seven *β*4GalT genes (*β*4GalT1 to 7) have been identified so far [22], [23]. To determine which *β*4GalT is associated with the aberrant glycosylation, *β*4GalT (1 to 7) mRNA expressions in purified B cells from *Smad4^co/co;Lck-cre^* mice and WT mice were analyzed by real-time RT-PCR using housekeeping GAPDH gene as the internal control. Real-time RT-PCR revealed that expression of mRNAs for *β*4GalT2 and 4 appeared to be significantly decreased in B-cells from *Smad4^co/co;Lck-cre^* mice as compared with B cells from WT mice (Figure 6).

## Discussion

Several reports indicate that, *in vitro* and *in vivo*, Th2 cytokines lead to increased production and defective glycosylation of IgA in the pathogenesis of IgAN [5]–[11], however, the molecular mechanisms are still unknown because appropriate animal model has not been established yet. In present study, T cell-specific *Smad4* deficient (*Smad4^co/co;Lck-cre^*) mice showed increased production of Th2 cytokines, high serum levels of IgA, develop mesangial IgA deposits associated with increased urinary albumin excretion, and dysregulation of IgA synthesis. This dysregulation manifested from increased serum IgA with aberrant glycosylation that includes an elevated proportion of polymer and that binds to IgG to form mixed isotype complexes. These features are commonly found with IgA isolated from patients with IgAN. Based on these results, *Smad4^co/co;Lck-cre^* mice could be a useful model to investigate the mechanisms by which the skewed Th2 cytokines result in the aberrant glycosylated IgA and IgA deposition for IgAN.

It has been well indicated that altered *O*-glycosylation of IgA hinge region leads IgA deposition and glomerular damage in IgAN. Meanwhile, murine IgA carries at least two *N*-linked oligosaccharide side chains in CH1 and CH3 domains but lacks *O*-linked ones. *β*4GalT transfer galactose from UDP-Gal to terminal *N*-acethylglucosamine (GlcNAc) of *N*-glycans in a *β*-1, 4 linkage to synthesize the Gal*β*1-4GlcNAc structure in murine IgA [22]. Recently, *β*4GalT1 deficient mice have been generated and showed IgAN-like phenotype such as completely lacking of sialyl galactose structure on IgA, mesangial IgA deposition, high serum IgA levels, and increased polymeric IgA [24]. Interestingly, our results demonstrated that mRNA levels of *β*4GalT2 and 4 were down-regulated in B cells from *Smad4^co/co;Lck-cre^* mice which was similar to *β*4GalT1 deficient mice in IgAN-like phenotype, as described above. These data suggest that not only *β*4GalT1 but also other *β*4GalTs have an important role in the terminal glycosylation of IgA, and skewed Th2 cytokines could be involved in altered *β*4GalT2 and 4 expression.

Smad4 is a key molecule that influences T cell differentiation; as part of a complex with Smad2 and Smad3, Smad4 serves as a transcription factor for forkhead box P3 (Foxp3), driving the expression of genes important for the generation of regulatory T cells (Tregs) [25]. Although the *Smad4^co/co;Lck-cre^* mice represent a useful model for the investigation of the balance between Th2 and Tregs, the means by which deletion of Smad4 promotes bias towards Th2 function remains unclear. A reciprocal relationship between Th2 cells and Tregs is recognized. The transcriptional activator STAT6 enhances the expression of the master regulator of Th2 differentiation, GATA3 [26]. STAT6 and GATA3 synergize with STAT5 activation to induce the secretion of Th2 cytokines by activated Th2 cells [27]. Apparently, Foxp3 can bind directly to GATA3; and engagement in this heterodimeric complex diminishes the activity of each binding partner [28], [29]. Although we did not assess the levels of Foxp3 and GATA3 expression in T cells from *Smad4^co/co;Lck-cre^* mice, the loss of *Smad4* would likely diminish Foxp3 expression, freeing GATA3 from inhibition while simultaneously impairing differentiation of Tregs. Indeed, a progressive decline in mucosal Treg has been described in the *Smad4^co/co;Lck-cre^* mice and in Smad3-/- mice [30]. This interpretation is also supported by a recent report showing that upon OVA challenge, glomerular IgA deposition was detected in only double-transgenic mice that overexpress GATA3 and the ovalbumin (OVA)-specific T cell receptor [6]. Assumingly, loss of *Smad4* gene in T cells, unopposed by Tregs, overproduction of Th2 cytokines, might down-regulate *β*4GalTs which are involved in glycosylation of IgA, and thus lead to exaggerated production of IgA that bears aberrant glycans.

The phenotype of *Smad4^co/co;Lck-cre^* mice in the present study is reminiscent of human IgAN and suggests that loss of Smad4 signaling in T cells associates with IgA deposition in the mesangial area, dysregulation of IgA, and proteinuria. However, there are some discrepancies between *Smad4^co/co;Lck-cre^* mice and spontaneous IgAN in humans. Notably, strong C3 deposition associated with mesangial IgA and hematuria are generally observed in human IgAN, but were not observed in *Smad4^co/co;Lck-cre^* mice. These discrepancies may represent requirements beyond glomerular IgA deposition, such as complement activation and/or inflammatory mediators, for evolution of hematuria. The pathogenesis of IgAN is well recognized as complicated and multifactorial [31], [32]. Recently, a genome-wide association study (GWAS) of human IgAN identified a major IgAN susceptibility locus within the complement factor H gene (CFH) cluster at chromosome 1q32 [33]. As CFH regulates complement activation through inhibition of the C3 convertase, the GWAS suggests that dysregulation of complement activation could be one of the mechanisms underlying progressive glomerular dysfunction. In addition to relatively minor glomerular complement deposition in spite of severe IgA deposition, the lack of hematuria and morphologically evident glomerular injury in *Smad4^co/co;Lck-cre^* mice may be explained by the strain of these knockout mice. According to reports in different murine models, glomerular dysfunction caused by IgA deposition is more pronounced in the BALB/c strain compared to other backgrounds despite similar mesangial deposition of IgA and C3 [20], [34], [35]. Although the basis for these strain differences is unclear, we expect that deletion of *Smad4* in BALB/c mice would lead to more morphologic and functional evidence of glomerular injury than observed in the current (C57BL/6×SvEv129×FVB) strain.

In our electron microscopic study, the patchy foot process effacement and no EDD were detected in glomeruli from *Smad4^co/co;Lck-cre^* mice. Most studies have reported that failure to detect EDD by electron microscopy is very rare in IgAN [36]–[38], in some studies absence of EDD by electron microscopy has been reported in up to 25% of cases [39]–[41]. The significance of EDD is still unclear, EDD is likely to be associated with clinical presentations of IgAN. Yoshikawa *et al*. have observed diminish of EDD by second biopsy of 23 IgAN patients with clinical remission [41], and we recently reported that serum albumin and estimated glomerular filtration rate in IgAN patients who had only paramesangial EDD were significantly lower than those in the patients who had EDD not only in paramesangial area but also in other areas [42]. In as yet unpublished data, we have observed foot process effacement without EDD at early phase in mice given injections of purified murine IgA. The foot process effacement could be easily occurred by IgA deposition before the formation of EDD, and *Smad4^co/co;Lck-cre^* mice might be most similar to slight-mild IgA nephropathy.

The source of nephritogenic IgA in IgAN (mucosal or bone marrow) is still controversial. Previous studies have implicated an association between secondary IgAN and inflammatory bowel disease (IBD) [43]–[45]. Transgenic mice which overexpress a ligand for lymphotoxin-β receptor (LIGHT) have been developed as animal model for IgAN [46]. This mouse model shows overproduction of IgA, an IgAN like-phenotype, severe intestinal inflammation, and other abnormalities in mucosal immunity, and implicates the intestine as the major source of glomerular IgA. In *Smad4^co/co;Lck-cre^* mice, it has been reported that the mice over 9 months of age also develop severe gastrointestinal inflammation, leading to spontaneous epithelial cancers throughout the gastrointestinal tract [15]. However, there are no distinct signs of intestinal inflammation in *Smad4^co/co;Lck-cre^* mice at 3 months of age despite of massive glomerular IgA deposition (Figure S1). Thus, bone marrow is likely to be the principal source of the increased IgA in our mouse model, and the mechanisms of IgA deposition into glomeruli in the mice are probably similar to that in human IgAN without IBD.

We conclude that *Smad4^co/co;Lck-cre^* mice produce heightened amounts of IgA that is mostly polymeric, aberrantly glycosylated, and complexed with IgG. These mice develop mesangial IgA deposits and proteinuria, reminiscent of human IgAN. *Smad4^co/co;Lck-cre^* mice could therefore provide us with new insights into the role that skewed Th2 cytokine production plays in altering circulating IgA. Ultimately, these insights might promote the development of a new therapeutic strategy for IgAN.

## Supporting Information

Figure S1 PAS-stained sections of small intestine and colon from both the *Smad4^co/co;Lck-cre^* and WT at 3 months of age. No histopathological abnormalities were observed. Original magnification; ×400
(PPTX)
