# Supplementary figures and images for: Disruption of Smad4 Expression in T Cells Leads to IgA Nephropathy-Like Manifestations
Source: PLoS One. 2013 Nov 4;8(11):e78736. doi: 10.1371/journal.pone.0078736 (PMC3817077; doi:10.1371/journal.pone.0078736)

## Slide 1
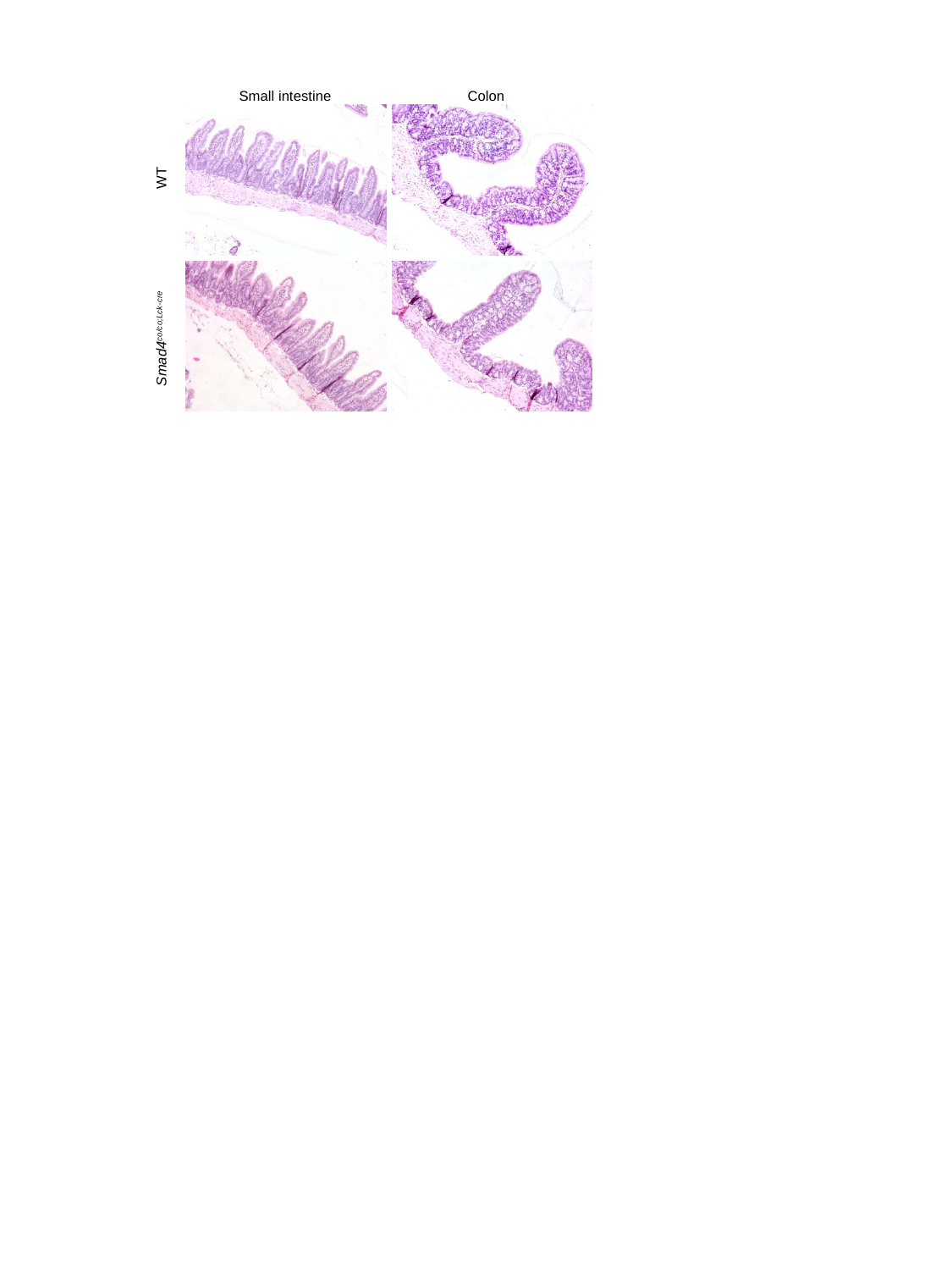

Small intestine
Colon
WT
Smad4co/co;Lck-cre

Supplement: Figure S1 — PAS-stained sections of small intestine and colon from both the Smad4co/co;Lck-cre and WT at 3 months of age. No histopathological abnormalities were observed. Original magnification; ×400 (PPTX) [file pone.0078736.s001.pptx]
